# Molecular Identification and Antifungal Susceptibility Patterns of *Candida* Species in Pediatric Patients at Tabriz Children′s Hospital

**DOI:** 10.1155/bmri/6685542

**Published:** 2026-06-05

**Authors:** Kamran Hosseini, Behrooz Naghili Hokmabadi, Narges Aslani, Taraneh Mirzai, Mahdieh Manafi Shabestari, Mohammad Ahangarzadeh Rezaee, Mohammad Bagher Hosseini, Azita Dilmaghani

**Affiliations:** ^1^ Shiraz Neuroscience Research Center, Shiraz University of Medical Sciences, Shiraz, Iran, sums.ac.ir; ^2^ Cardiovascular Research Center, Shiraz University of Medical Sciences, Shiraz, Iran, sums.ac.ir; ^3^ Infectious and Tropical Diseases Research Center, Tabriz University of Medical Sciences, Tabriz, Iran, tbzmed.ac.ir; ^4^ Department of Parasitology and Mycology, School of Medicine, Urmia University of Medical Sciences, Urmia, Iran, umsu.ac.ir; ^5^ Department of Pharmaceutical Biotechnology, Faculty of Pharmacy, Tabriz University of Medical Sciences, Tabriz, Iran, tbzmed.ac.ir; ^6^ Pediatric Health Research Center, Tabriz University of Medical Science, Tabriz, Iran, tbzmed.ac.ir; ^7^ Department of Microbiology, Faculty of Medicine, Tabriz University of Medical Sciences, Tabriz, Iran, tbzmed.ac.ir

**Keywords:** antifungal susceptibility, broth microdilution, *C. albicans*, *Candida* species, drug resistance, ITS sequencing, pediatric candidiasis

## Abstract

*Candida* species are significant opportunistic pathogens, particularly in immunocompromised pediatric populations. With the increasing incidence of invasive candidiasis in neonatal and pediatric intensive care units, accurate species identification and antifungal susceptibility profiling have become critical for effective treatment. This dual‐phase study is aimed at: (1) identifying *Candida* species from clinical samples using conventional and molecular methods (rDNA‐ITS sequencing), and (2) evaluating antifungal susceptibility patterns against fluconazole, itraconazole, amphotericin B, and caspofungin. A total of 97 clinical isolates were obtained from pediatric patients for molecular identification, with 67 isolates successfully subcultured and evaluated for antifungal susceptibility testing. Species identification involved culture on selective media, microscopic examination, DNA extraction, and ITS region sequencing. Antifungal susceptibility testing was performed using the broth microdilution method (CLSI M27 standard) with RPMI‐1640 medium, incubating at 35°C for 48 h. Minimum inhibitory concentration (MIC) endpoints were determined visually. Molecular identification revealed the following distribution: *C. glabrata* (50 isolates, 51.5%), *C. albicans*/*dubliniensis* complex (34, 35.1%), *C. kefyr* (5, 5.2%), *C. krusei* (6, 6.2%), and *C. tropicalis* (2, 2.1%). Susceptibility testing showed widespread resistance to fluconazole (particularly in *C. glabrata*, *C. albicans*, and *C. krusei*) and amphotericin B; variable resistance to itraconazole (with 100% susceptibility in *C. kefyr*); and universal susceptibility to caspofungin (97.4%–100% across species). Our findings highlight a concerning epidemiological shift toward nonalbicans species, predominantly *C. glabrata*, with distinct resistance profiles. While caspofungin remains highly effective, the observed resistance patterns underscore the necessity of routine antifungal susceptibility testing to guide therapy and prevent treatment failure in pediatric candidiasis.

## 1. Introduction

Nosocomial infections are infections that infants and hospitalized patients acquire during their hospital stay, with symptoms appearing either during hospitalization or after discharge [1]. Many pathogens are involved in bacterial, viral, and fungal nosocomial infections. The most common pathogens include *Staphylococcus*, *Pseudomonas*, *Escherichia coli*, *Acinetobacter*, *Candida*, *Aspergillus*, *Fusarium*, *Trichosporon*, and *Malassezia* genera [2]. According to numerous studies, *Candida* yeast was identified as the fourth most common cause of hospital‐acquired infections [3].

The genus *Candida* consists of about 200 species, which exist as single cells and reproduce by budding [4]. These single‐celled yeasts produce infections that range from non–life‐threatening mucocutaneous diseases to invasive processes that may involve almost any organ in the body [5]. Over the past two decades, invasive candidiasis (IC) has increased in neonates admitted to intensive care units, especially premature infants, and has been associated with significant mortality (30%–75%) [6]. Research shows that 7%–20% of all premature infants develop disseminated infection with this fungus following *Candida* colonization [7].

Diagnosis of *Candida* infections depends on the location of the infections, the symptoms observed, microscopic examination, culture of clinical specimens, morphological identification, rapid identification tests such as enzymatic and immunological methods, biochemical characterization such as sugar auxanogram examination, and molecular identification such as PCR, karyotyping, MLP typing, and DNA microarray [8]. Molecular techniques, particularly PCR targeting the rDNA‐ITS region, have emerged as precise, rapid, and reliable tools for *Candida* species identification [9].

Antifungal prophylaxis is an effective preventive strategy in other pediatric populations at risk for candidemia, including neonates and oncology patients [10]. Antifungal resistance of *Candida* species is a clinical problem in the management of diseases caused by these pathogens [11]. The development of antifungal resistance is complex and depends on multiple host and microbial factors [12]. One of the specific pathogenic features of *Candida* species is their ability to form biofilms, which protect them from external factors such as the host defense system and antifungal drugs [13]. Biofilms are genetically resistant to antifungal agents, including amphotericin B and fluconazole. Biofilm formation varies depending on the *Candida* species [14]. Recently, *Candida albicans* (*C. albicans*) has emerged as the predominant species in IC, and a relative shift in nonalbicans species such as *Candida glabrata* (*C. glabrata*) and *Candida krusei* (*C. krusei*) has been observed with increasing resistance to antifungal drugs. Therefore, identification of *Candida* species and antifungal drug susceptibility testing are very important for selecting appropriate treatment [15].

This study was designed in two phases: (1) molecular characterization of 97 Candida isolates from neonates and children at Tabriz Children′s Hospital using rDNA‐ITS PCR, and (2) evaluation of antifungal susceptibility patterns in 67 viable isolates to guide treatment strategies. Therefore, the specific aims of this study were to: (1) accurately identify *Candida* species isolated from pediatric patients at Tabriz Children′s Hospital using molecular methods (rDNA‐ITS sequencing); (2) determine the species distribution and demographic characteristics of infected patients; (3) evaluate the in vitro susceptibility patterns of the identified isolates to four commonly used antifungal agents (fluconazole, itraconazole, amphotericin B, and caspofungin); and (4) compare the efficacy of molecular identification methods with conventional phenotypic approaches for species‐level characterization. Our findings are expected to provide critical insights for infection control and therapeutic decision‐making in this vulnerable population.

## 2. Materials and Methods

### 2.1. Sample Collection

**Figure 1:**
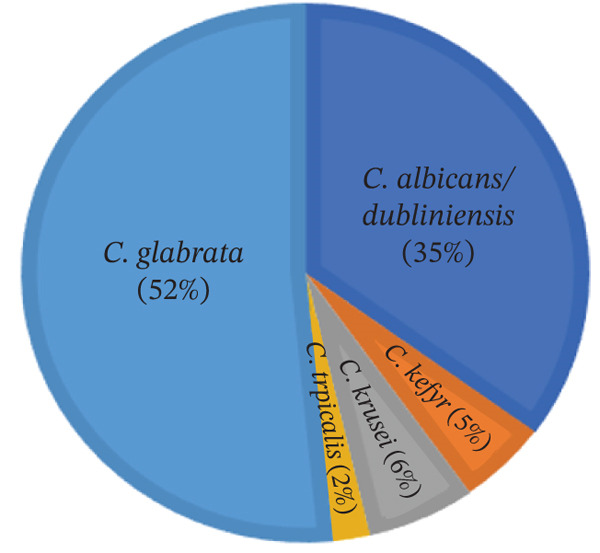
Distribution of *Candida* species isolated from pediatric patients (*n* = 97). Each slice shows the species name and corresponding percentage. The most prevalent species was *C. glabrata* (52%), followed by the *C. albicans*/*dubliniensis* complex (35%), *C. krusei* (6%), *C. kefyr* (5%), and *C. tropicalis* (2%).

*Candida* isolates previously obtained from pediatric patients hospitalized at Tabriz Children′s Hospital were incorporated into this study. These isolates were recovered from diverse clinical specimens collected as part of routine diagnostic protocols. It should be noted that while a total of 97 clinical isolates were collected for molecular identification, only 67 isolates could be successfully recovered and subcultured for antifungal susceptibility testing. The remaining 30 isolates were either nonviable upon subculture or could not be propagated sufficiently to meet the inoculum requirements for standardized broth microdilution testing as per CLSI guidelines. Clinical isolates were collected over a 12‐month period from March 2021 to February 2022 from pediatric patients hospitalized at Tabriz Children′s Hospital. Patients were admitted to various hospital units including: neonatal intensive care unit (NICU), pediatric intensive care unit (PICU), infectious diseases ward, internal medicine wards (A, B, and C), operating room, and the hematology–oncology ward. The distribution of isolates across these units is presented in Figure 1 and Table 1. Clinical specimens included both invasive and noninvasive samples obtained as part of routine diagnostic procedures. The following sample types were collected: urine (*n* = 44), wound swabs (*n* = 37), cerebrospinal fluid shunt specimens (*n* = 5), catheter tips (*n* = 4), peritoneal fluid (*n* = 3), blood cultures (*n* = 3), and one cerebrospinal fluid sample (*n* = 1). Invasive samples (blood, cerebrospinal fluid, peritoneal fluid, and catheter tips) constituted approximately 15% of total specimens, whereas noninvasive samples (urine and wound swabs) comprised the remaining 85%. All samples were collected under sterile conditions and processed immediately upon arrival at the medical mycology laboratory.

**Table 1:** Culture results on CHROMagar medium, color, and type of *Candida* species.

No.	Color	*Candida* species	Genus (male or female)	Age	Sample source	Ward	Size of ITS1 or ITS4	Germinal tube test result
N 90a	White	*C. glabrata*	M	1 month	Wound	NICU	871	—
P 11b	Green	*C. albicans* or *C. dubliniensis*	M	4 years	Catheter	Operating room	535	+
P 9a	Green	*C. albicans* or *C. dubliniensis*	M	8 years	Cerebrospinal fluid shunt	PICU	535	+
N 1a	Pink	*C. kefyr*	M	2 years	Urine	Infectious	748	—
N 13	Pink	*C. kefyr*	M	1 year	Urine	Internal Ward B	748	—
NA 81a	Green	*C. albicans* or *C. dubliniensis*	F	23 months	Wound	Operating room	535	+
N 46a	White	*C. glabrata*	M	2 years	Urine	PICU	871	—
P 9C	Blue	*C. tropicalis*	M	8 years	Cerebrospinal fluid shunt	PICU	524	+
N 74	White	*C. glabrata*	M	3 years	Wound	Infectious	871	—
N 73	Pink	*C. kefyr*	F	6 months	Wound	Internal Ward B	748	—
N 79b1	White	*C. glabrata*	F	22 months	Wound	Infectious	871	—
NA 1	Pink	*C. kefyr*	M	3 years	Urine	PICU	748	—
N 72b	White	*C. glabrata*	M	6 months	Urine	Infectious	871	—
P 4	Green	*C. albicans* or *C. dubliniensis*	M	3 days	Urine	NICU	535	+
N 82c	Green	*C. albicans* or *C. dubliniensis*	M	2 years	Cerebrospinal fluid shunt	Operating room	535	+
N 89b	Pink	*C. krusei*	M	21 months	Urine	NICU	510	—
N 69b	White	*C. glabrata*	F	5 years	Urine	Infectious	871	—
N 68	White	*C. glabrata*	M	6 months	Wound	NICU	871	—
P 15	Green	*C. albicans* or *C. dubliniensis*	M	2 months	Urine	Infectious	535	+
N 80a	White	*C. glabrata*	F	2 years	Peritonitis fluid	Infectious	871	—
N 80b	White	*C. glabrata*	M	7 years	Urine	Internal Ward B	871	—
N 82b	Green	*C. albicans* or *C. dubliniensis*	M	4 months	Urine	PICU	535	+
NA 81c	Green	*C. albicans* or *C. dubliniensis*	M	3 years	Urine	PICU	535	+
N 67b	Green	*C. albicans* or *C. dubliniensis*	M	8 years	Peritonitis fluid	Operating room	535	+
N 68b	White	*C. glabrata*	F	11 months	Wound	Infectious	871	—
N 69a	White	*C. glabrata*	F	3 months	Wound	Internal Ward A	871	—
N 71C	White	*C. glabrata*	F	3 years	Wound	Infectious	871	—
N 44a	White	*C. glabrata*	M	4 years	Wound	Internal Ward A	871	—
NA 12	White	*C. glabrata*	F	1 year	Wound	Operating room	871	—
N 72a	White	*C. glabrata*	M	5 years	Urine	Infectious	871	—
N 1b	Pink	*C. krusei*	M	2 months	Urine	NICU	510	—
N 79b2	White	*C. glabrata*	M	3 years	Urine	Infectious	871	—
N 90c	Pink	*C. kefyr*	F	4 years	Wound	Internal Ward A	748	+
N 64b	White	*C. glabrata*	F	8 months	Wound	Infectious	871	—
NA 81b	Green	*C. albicans* or *C. dubliniensis*	M	3 years	Catheter	PICU	535	+
NA 5b	White	*C. glabrata*	M	2 years	Urine	PICU	871	—
N 81a	Green	*C. albicans* or *C. dubliniensis*	M	4 years	Urine	Internal Ward B	535	+
NA 82a	Green	*C. albicans* or *C. dubliniensis*	M	4 months	Urine	Infectious	535	+
P 9b	Green	*C. albicans* or *C. dubliniensis*	M	8 years	Cerebrospinal fluid shunt	Infectious	535	+
NA 78	White	*C. glabrata*	M	2 years	Wound	Infectious	871	—
N 51b	White	*C. glabrata*	F	6 months	Wound	PICU	871	—
N 84	Green	*C. albicans* or *C. dubliniensis*	F	8 years	Wound	Infectious	535	+
N 89a	White	*C. glabrata*	F	3 months	Catheter	NICU	871	—
N 18	White	*C. glabrata*	M	5 years	Wound	Infectious	871	—
N 85a	Green	*C. albicans* or *C. dubliniensis*	M	8 years	Wound	Operating room	535	+
N 12	White	*C. glabrata*	M	1 year	Wound	Internal Ward A	871	—
N 5	White	*C. glabrata*	M	3 months	Urine	Internal Ward C	871	—
NA 82b	Green	*C. albicans* or *C. dubliniensis*	M	8 months	Peritonitis fluid	Infectious	535	+
N 90a	White	*C. glabrata*	M	11 months	Urine	Internal Ward C	871	—
N 45a	White	*C. glabrata*	F	4 months	Urine	NICU	871	—
NA 67	Green	*C. albicans* or *C. dubliniensis*	M	6 years	Urine	Blood	535	+
NA 21a	Green	*C. albicans* or *C. dubliniensis*	F	7 months	Cerebrospinal fluid shunt	Infectious	535	+
N 51a	White	*C. glabrata*	F	2 years	Wound	PICU	871	—
N 70a	White	*C. glabrata*	M	9 years	Wound	Infectious	871	—
NA 21b	Green	*C. albicans* or *C. dubliniensis*	M	4 months	Urine	NICU	535	+
N 11	White	*C. glabrata*	F	7 months	Urine	PICU	871	—
N 7	White	*C. glabrata*	M	8 years	Catheter	Blood	871	—
N 64a	White	*C. glabrata*	M	1 year	Urine	Infectious	871	—
N 70b	White	*C. glabrata*	M	4 years	Urine	Infectious	871	—
N 44b	White	*C. glabrata*	F	4 years	Wound	Infectious	871	—
N 71a	White	*C. glabrata*	M	7 months	Urine	Operating room	871	—
N 84b	Green	*C. albicans* or *C. dubliniensis*	F	1 month	Wound	NICU	535	+
N 80b	White	*C. glabrata*	F	8 months	Wound	Operating room	871	—
P 9b	Green	*C. albicans* or *C. dubliniensis*	M	8 years	Cerebrospinal fluid shunt	PICU	535	+
N 90a	Pink	*C. krusei*	M	3 years	Urine	Infectious	510	—
N 45b	White	*C. glabrata*	F	7 years	Urine	Infectious	871	—
N 81	Green	*C. albicans* or *C. dubliniensis*	F	1 year	Urine	Blood	535	+
P 11a	Green	*C. albicans* or *C. dubliniensis*	M	4 years	Catheter	Operating room	535	+
NA 71a	White	*C. glabrata*	F	2 years	Wound	Infectious	871	—
N 84a	Green	*C. albicans* or *C. dubliniensis*	F	1 month	Wound	Infectious	535	+
NA 71b	Pink	*C. krusei*	F	7 months	Wound	Infectious	510	—
P 11b	Green	*C. albicans* or *C. dubliniensis*	M	4 years	Catheter	Operating room	535	+
N 71b	White	*C. glabrata*	M	5 years	Urine	Infectious	871	—
77a	White	*C. glabrata*	M	8 years	Urine	Infectious	871	—
N 42	White	*C. glabrata*	M	9 months	Urine	Infectious	871	—
P 13	Green	*C. albicans* or *C. dubliniensis*	M	1 month	Urine	Internal Ward A	535	+
N 82a	Green	*C. albicans* or *C. dubliniensis*	F	2 years	Wound	Infectious	535	+
P 10	Green	*C. albicans* or *C. dubliniensis*	M	4 years	Blood culture	Internal Ward C	535	+
NA 5a	White	*C. glabrata*	F	4 years	Wound	Blood	535	—
P 2a	Green	*C. albicans* or *C. dubliniensis*	M	7 years	Urine	Blood	5535	+
N 80a	White	*C. glabrata*	F	3 months	Urine	NICU	871	—
N 90b	Pink	*C. krusei*	F	2 years	Urine	Internal Ward B	510	—
N 75a	White	*C. glabrata*	M	8 years	Urine	Internal Ward C	871	—
N 78a	Pink	*C. krusei*	M	3 years	Urine	Internal Ward C	510	—
N 72a	White	*C. glabrata*	M	4 months	Urine	NICU	810	—
N 78b	White	*C. glabrata*	M	6 years	Urine	Blood	810	—
P 14	Green	*C. albicans* or *C. dubliniensis*	M	1 month	Urine	Internal Ward A	535	+
N 85b	Green	*C. albicans* or *C. dubliniensis*	M	6 months	Wound	Infectious	535	+
P 2b	Green	*C. albicans* or *C. dubliniensis*	M	7 years	Urine	Blood	535	+
N 74	White	*C. glabrata*	F	5 years	Wound	Internal Ward B	871	—
P 8	Green	*C. albicans* or *C. dubliniensis*	F	1 month	Urine	NICU	535	+
N 84b	Blue	*C. tropicalis*	M	6 years	Wound	Internal Ward A	524	—
N 77b	White	*C. glabrata*	M	8 years	Wound	Internal Ward B	871	—
N 84c	White	*C. glabrata*	M	8 months	Wound	Internal Ward B	871	—
N 78b	White	*C. glabrata*	M	9 years	Wound	Infectious	871	—
N 89c	Green	*C. albicans* or *C. dubliniensis*	M	1 month	Wound	Infectious	535	+
N 72b	White	*C. glabrata*	F	2 years	Wound	Infectious	871	—

### 2.2. Culture and Isolation

All clinical specimens were cultured on Sabouraud Dextrose Agar (SDA) supplemented with chloramphenicol (0.05 g/L) to inhibit bacterial growth. After medium preparation, clinical samples were streaked onto the prepared plates using a four‐quadrant streaking technique to obtain isolated colonies. After incubation, distinct yeast colonies were selected and subcultured on fresh SDA plates to obtain pure cultures for further identification and characterization. Pure colonies were maintained on SDA slants at 4°C for short‐term storage and in Tryptic Soy Broth with 20% glycerol at −20°C for long‐term preservation.

### 2.3. Chromogenic Agar Identification

Following initial growth on SDA, yeast‐like colonies were presumptively identified based on colony morphology, Gram staining characteristics (gram‐positive, oval‐to‐round budding yeast cells), and microscopic examination. Colonies exhibiting typical yeast morphology were then subcultured on CHROMagar *Candida* plates for presumptive species identification. CHROMagar *Candida* (CHROMagar, Paris, France) is a selective differential culture medium that contains chromogenic substrates. Different *Candida* species produce distinct colony colors due to species‐specific enzyme activities that react with the chromogenic substrates in the medium. CHROMagar plates were prepared by dissolving the commercial powder in 265‐mL sterile distilled water according to the manufacturer′s instructions. Fresh subcultures were streaked in four quadrants and incubated at 37°C for 48 h. Species were presumptively identified based on distinctive colony pigmentation: *C. albicans* and *Candida dubliniensis* (*C. dubliniensis*) developed green coloration, *Candida tropicalis* (*C. tropicalis*) appeared as blue colonies, *C. krusei* and *Candida kefyr* (*C. kefyr*) produced pink hues, whereas *C. glabrata* formed white colonies. All color interpretations were made under natural light against a white background, with photographic documentation of characteristic morphologies for reference. This chromogenic method provided rapid preliminary identification within 24–48 h while simultaneously enabling detection of mixed cultures through differential coloration of coexisting species.

### 2.4. Germinal Tube Test for *C. albicans* Identification

To confirm *C. albicans*, the germinal tube test was performed on well‐isolated colonies (24–48‐h growth on SDA). Colonies were emulsified in 0.5 mL of sterile bovine serum using a sterile loop to form a slightly turbid suspension. This mixture was transferred to a sterile microtube and incubated at 37°C in a water bath for exactly 2 h. After incubation, a small drop of the suspension was placed on a clean glass slide, covered with a coverslip, and immediately examined under 40× magnification using a light microscope. The formation of germinal tubes (characteristic hyphal‐like extensions without constrictions at their point of origin from the yeast cell) within this timeframe was considered positive for *C. albicans* identification. All microscopic examinations were performed by two independent observers to minimize interpretation errors.

### 2.5. DNA Extraction and PCR Amplification

DNA was extracted from pure *Candida* colonies using a modified boiling method. Briefly, one to two colonies were suspended in 100‐*μ*L lysis buffer (10 mM Tris‐HCl, 1 mM EDTA, 1% SDS, 2% Triton X‐100, 100 mM NaCl) and vortexed vigorously. The suspension was boiled at 100°C for 15 min, then mixed with 100 *μ*L of 2.5 M sodium acetate and placed at −20°C for 1 h. After centrifugation (12,000 × g, 5 min), the supernatant was transferred to a new tube and mixed with an equal volume of cold isopropanol. Following incubation at −20°C for 30 min and centrifugation (10,000 × g, 15 min), the DNA pellet was washed with 75% and 99% ethanol, air‐dried, and resuspended in 50‐*μ*L distilled water. DNA concentration was measured spectrophotometrically at 260 nm.

For PCR amplification, 25 *μ*L reactions contained 12.5 *μ*L master mix, 1 *μ*L each of ITS1 (5 ^′^‐TCCGTAGGTGAACCTGCGG‐3 ^′^) and ITS4 (5 ^′^‐TCCTCCGCTTATTGATATGC‐3 ^′^) primers (10 pmol/*μ*L), 1 *μ*L DNA template, and 9.5 *μ*L nuclease‐free water. Amplification conditions included: initial denaturation at 94°C for 5 min; 35 cycles of 94°C for 30 s, 56°C for 45 s, 72°C for 1 min; and final extension at 72°C for 7 min. PCR products were analyzed by 1.5% agarose gel electrophoresis at 90 V for 30 min and visualized under UV illumination.

### 2.6. Antifungal Susceptibility Testing

Antifungal susceptibility testing was performed using the broth microdilution method according to the Clinical and Laboratory Standards Institute (CLSI) M27‐A3 and M27‐S4 guidelines. Stock solutions of fluconazole (Table 2), itraconazole, amphotericin B, and caspofungin (Table 3) were prepared in dimethyl sulfoxide (DMSO) and stored at −80°C until use. Serial twofold dilutions of each antifungal agent were prepared in RPMI‐1640 medium (with L‐glutamine, without bicarbonate, buffered to pH 7.0 with MOPS) to achieve final concentration ranges of 0.0625–128 *μ*g/mL for fluconazole and 0.0625–32 *μ*g/mL for itraconazole, amphotericin B, and caspofungin.

**Table 2:** Method for preparing fluconazole dilutions.

**Level**	**Concentration mL/*μ*g**	**Source**	**Volume (mL)**	**Medium of RPMI 1640 (mL)**	**Middle conc. mL/*μ*g**	**Final conc. 1:5 mL/*μ*g**
1	5120	Stock	1	7	640	128
2	640	Level 1	1	7	320	64
3	640	Level 1	1	3	160	32
4	160	Level 3	1	1	80	16
5	160	Level 3	0.5	1.5	40	8
6	160	Level 3	0.5	3.5	20	4
7	20	Level 6	1	1	10	2
8	20	Level 6	0.5	1.5	5	1
9	20	Level 6	0.5	3.5	2.5	0.5
10	2.5	Level 9	1	1	1.25	0.25
11	2.5	Level 9	0.5	1.5	0.625	0.125
12	2.5	Level 9	0.5	3.5	0.3125	0.0625

**Table 3:** Method for preparing dilutions of itraconazole, caspofungin, and amphotericin B.

**Level**	**Concentration mL/*μ*g**	**Resource**	**Volume (mL)**	**DMSO (mL)**	**Middle conc. mL/*μ*g**	**Final conc. 1:50 mL/*μ*g**
1	1600	Stock	—	—	1600	32
2	1600	Stock	0.5	0.5	800	16
3	1600	Stock	0.5	1.5	400	8
4	1600	Stock	0.5	3.5	200	4
5	200	Level 4	0.5	0.5	100	5
6	200	Level 4	0.5	1.5	50	4
7	200	Level 4	0.5	3.5	25	0.5
8	25	Level 7	0.5	0.5	12.5	0.25
9	25	Level 7	0.5	1.5	6.25	0.125
10	25	Level 7	0.5	3.5	3.125	0.0625

Yeast inocula were prepared from 48‐h cultures on SDA and adjusted spectrophotometrically to a concentration of 0.5 × 10^3^ to 2.5 × 10^3^ CFU/mL in RPMI‐1640 medium. Microplates were incubated at 35°C for 48 h. The minimum inhibitory concentration (MIC) was defined as the lowest drug concentration that resulted in complete inhibition of growth (for amphotericin B and caspofungin) or ≥ 50% reduction in growth compared with the drug‐free control (for fluconazole and itraconazole). *Candida parapsilosis* ATCC 22019 and *C. krusei* ATCC 6258 were included as quality control strains in each testing run. All tests were performed in duplicate.

### 2.7. Performing the Broth Microdilution Test

Serial dilutions of the drugs were prepared according to Tables 2 and 3. A total of 100 *μ*L of a twofold concentration of the desired drug was distributed into Wells 1–11 of a 96‐well microplate. The first well contained the highest concentration of the drug, and Well 11 contained the lowest concentration of the drug. Then, 100 *μ*L of a twofold concentration of yeast cell suspension was added to each well, and the two were diluted 1:1 to reach the required final concentration. Well 12, or the drug‐free growth control, was considered as the well with zero drug concentration. This well contained 100 *μ*L of drug‐free culture medium and 100 *μ*L of yeast suspension and was used as a criterion for comparing growth with other wells. The microplates were incubated at 35°C for 48 h. To expedite the work, 96‐well microplates containing the aforementioned drug dilutions were prepared and stored in the freezer until use without deterioration or any changes in the drugs, and on the day of the test, only the yeast suspension under test was prepared and added to them. Similar steps were taken from preparing the yeast suspension to testing for each of the reference isolates, and the results obtained were used as a standard to judge the determination of the MIC of the isolated samples to the drugs under test.

### 2.8. Reading MIC Results

MIC endpoints were determined visually after 48 h of incubation at 35°C. The interpretation criteria differed by antifungal class according to CLSI guidelines: For amphotericin B and caspofungin, the MIC was defined as the lowest concentration causing complete inhibition of visible growth (100% inhibition). For fluconazole and itraconazole, the MIC was defined as the lowest concentration producing a prominent decrease in turbidity (≥ 50% inhibition) compared with the drug‐free growth control. All readings were performed against a white background with adequate illumination using a convex mirror reading device. Susceptibility categories (susceptible, intermediate, and resistant) were assigned based on CLSI clinical breakpoints when available, or epidemiological cutoff values when clinical breakpoints were not defined.

### 2.9. Molecular Identification by ITS (Internal Transcribed Spacer) Sequencing

Following PCR amplification with ITS1 and ITS4 primers, amplicons were visualized by agarose gel electrophoresis to confirm successful amplification and estimate fragment size. Although ITS region length polymorphisms can provide preliminary species indication (e.g., *C. albicans* ~535 bp, C. glabrata ~871 bp), fragment size alone is insufficient for definitive species identification due to intraspecies variability and the possibility of comigrating fragments from different species. Therefore, for definitive species identification, all PCR products were purified using a commercial PCR purification kit (Qiagen, Germany) and subjected to bidirectional DNA sequencing using the same ITS1 and ITS4 primers. Sequencing was performed on an ABI 3730xl DNA Analyzer (Applied Biosystems, United States).

The resulting sequences were analyzed using Chromas software (Version 2.6.6) for quality assessment and manual editing of ambiguous bases. Consensus sequences were compared against reference sequences in the GenBank database using the BLAST algorithm (https://blast.ncbi.nlm.nih.gov). Species assignment was based on the following criteria: ≥ 99% sequence identity with a reference strain in GenBank, query coverage ≥ 98%, E‐value ≤ 1e‐50, and the top BLAST hit with a clearly defined species identification.

## 3. Results

### 3.1. Results of Cultivation on *Candida* Chrome Agar Medium

**Figure 2:**
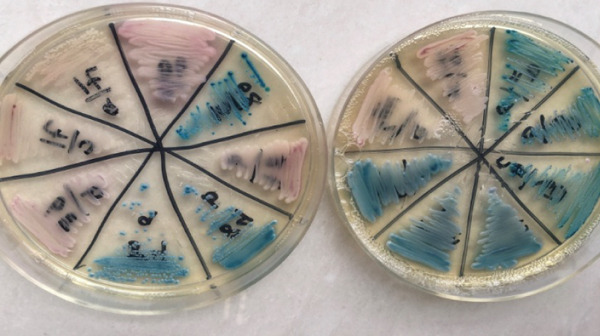
Colony color of *Candida* species on *Candida* chrome agar medium.

Chromogenic agar medium was used to confirm the identity of the *Candida* species. Ninety‐seven *Candida* species produced green, white, blue, and pink colors on this medium. According to Figure 2, pink color corresponds to *C. krusei* and *C. kefyr*, green color corresponds to *C. albicans* and *C. dubliniensis*, blue color corresponds to *C. tropicalis*, and white color corresponds to *C. glabrata*.

As shown in Figure 1, which presents the species distribution with labeled percentages, *C. glabrata* was the most prevalent species (52%, *n* = 50), whereas *C. tropicalis* was the least common (2%, *n* = 2). The *C. albicans*/*dubliniensis* complex accounted for 35% of isolates (*n* = 34), followed by *C. krusei* (6%, *n* = 6) and *C. kefyr* (5%, n = 5). As can be seen in Table 1, samples were selected from both male and female sexes in the age range of 3 days to 9 years. Most of the patients′ urine and wound samples were used for sampling.

**Figure 3:**
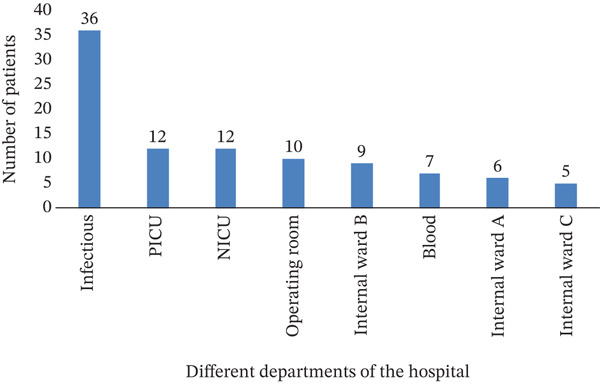
Distribution chart of *Candida* species in different hospital departments.

Among the 44 urine samples, the highest frequency was related to *C. glabrata* with 22 cases (50%), followed by *C. albicans* and *C. dubliniensis* with 14 cases (31%); *C. tropicalis* was not detected in any urine samples. Of the 37 wound samples, *C. glabrata* had the highest frequency with 25 cases (67%), and *C. krusei* and *C. tropicalis* had the lowest frequency with one case (0.027%). As can be seen in Table 1, germinal tubes were formed in the samples containing *C. albicans* and *C. dubliniensis.* In addition, according to Figure 3, the most likely area for the presence of *Candida* species is the hospital′s infectious department, and *Candida* species are less likely to be present in Internal Ward C.

Among the 97 clinical isolates collected from pediatric patients aged between 3 days and 9 years, *C. glabrata* was the most frequently identified species in the molecular identification phase. Of these, 67 isolates (38 *C. glabrata*, 20 *C. albicans*, 4 *C. krusei*, 4 *C. kefyr*, and 1 *C. tropicalis*) were successfully subcultured and included in the antifungal susceptibility testing phase. It was characterized by white colonies, an ITS region size of approximately 871 bp, and consistently negative germinal tube formation. The second most prevalent species was *C. albicans*/*dubliniensis*, distinguished by green colonies, an ITS size of approximately 535 bp, and uniformly positive germinal tube results—an important diagnostic feature that differentiates it from other *Candida* species.

Less commonly detected isolates included *C. kefyr* (pink colonies, ~748 bp, germinal tube negative), *C. krusei* (pink colonies, ~510 bp), and *C. tropicalis* (blue colonies, ~524 bp), which were rarely observed. The majority of isolates were obtained from urine and wound samples, followed by cerebrospinal fluid shunts, catheters, peritoneal fluid, and blood specimens.

In terms of hospital settings, the infectious disease ward, NICU, and PICU were the primary sources of isolates, reflecting their critical role in managing high‐risk, immunocompromised pediatric patients. Male patients were slightly more represented than females. Age distribution analysis revealed a higher incidence among infants and toddlers, particularly those under 2 years of age—likely due to their immature immune systems and increased exposure to invasive medical devices.

This dataset underscores the diagnostic value of integrating colony morphology, ITS region sizing, and germinal tube testing for accurate identification of *Candida* species. Such precision is essential for guiding effective antifungal therapy and implementing targeted infection control strategies in pediatric healthcare settings. In addition, according to Figure 3, the most likely area for the presence of *Candida* species is the hospital′s infectious disease department. In the Internal C department, *Candida* species are less likely to be present.

### 3.2. Germinal Tube Test Results

**Figure 4:**
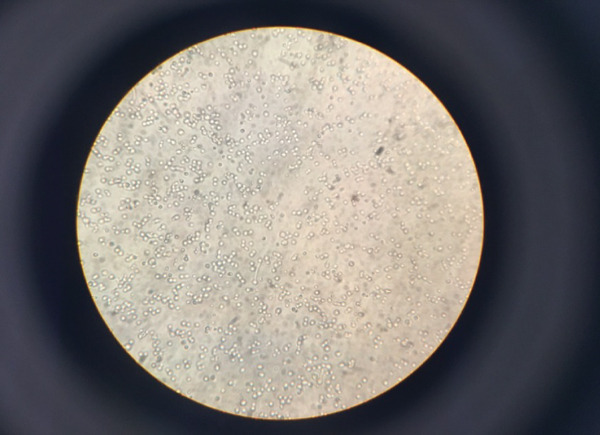
Results of germinal tube examination under a light microscope.

To perform this test, bovine serum and a sample containing *Candida* are mixed. This test is used to differentiate *C. albicans* from other *Candida* species. In Figure 4, an example of germinal tube formation was observed under the microscope, indicating the presence of *C. albicans* and *C. dubliniensis* species in the samples.

### 3.3. PCR Reaction Results

**Figure 5:**
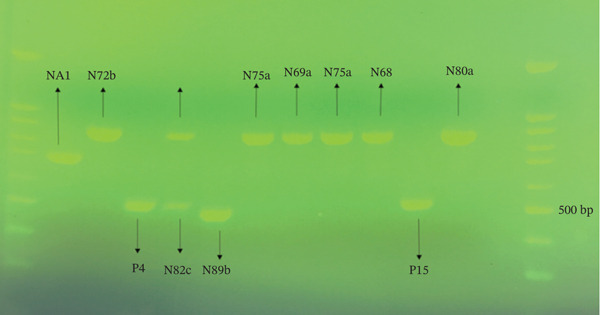
Electrophoresis of PCR products of different *Candida* species using ITS1–ITS4 primers.

To amplify the extracted DNA products, PCR reactions were performed on 97 candidates. PCR amplification of the ITS region yielded products of varying sizes: approximately 535 bp for the *C. albicans*/*dubliniensis* complex, 871 bp for *C. glabrata*, 748 bp for *C. kefyr*, 510 bp for *C. krusei*, and 524 bp for *C. tropicalis*. These size differences provided initial orientation; however, definitive species identification was achieved through DNA sequencing and BLAST analysis as described in Methods. Representative electrophoresis results are shown in Figure 5.

Overall, the observed species distribution emphasizes the clinical relevance of implementing molecular diagnostic tools for rapid and precise identification of *Candida* species. Such approaches are essential for optimizing patient management, particularly in immunocompromised individuals and those in intensive care settings.

#### 3.3.1. Results of the Test to Measure the Organism′s Susceptibility to Antifungal Drugs

In this study, as mentioned, the standard broth microdilution method was used as provided in CLSI‐M27‐A3. To investigate the MIC, each of the studied drugs, namely fluconazole, itraconazole, amphotericin B, and caspofungin, was used. Table 4 shows the MIC results of four antifungal drugs for 67 *Candida* isolates. Antifungal susceptibility testing was successfully performed on 67 of the 97 original isolates. Table 4 presents the MIC results of four antifungal drugs for these 67 *Candida* isolates.

**Table 4:** MIC results of four antifungal drugs for 67 *Candida* isolates that underwent susceptibility testing.

MIC *μ*g/mL				Species
Fluconazole	Itraconazole	Amphotericin B	Caspofungin	
16	4	8	0.007	*C. glabrata*
64	8	4	0.007	*C. glabrata*
64	8	16	2	*C. glabrata*
64	8	8	0.007	*C. glabrata*
64	8	16	0.007	*C. glabrata*
64	16	16	0.007	*C. glabrata*
64	16	16	0.007	*C. glabrata*
64	2	16	0.007	*C. glabrata*
64	0.03	16	0.007	*C. glabrata*
64	4	16	0.007	*C. glabrata*
64	8	16	0.007	*C. glabrata*
64	8	16	0.007	*C. glabrata*
64	8	16	0.007	*C. glabrata*
64	4	16	0.007	*C. glabrata*
64	16	16	0.007	*C. glabrata*
64	2	16	0.007	*C. glabrata*
64	1	16	0.007	*C. glabrata*
64	2	16	0.007	*C. glabrata*
64	4	16	0.007	*C. glabrata*
64	4	16	0.007	*C. glabrata*
64	0. 5	16	0.007	*C. glabrata*
64	2	16	0.007	*C. glabrata*
64	1	16	0.007	*C. glabrata*
32	16	8	0.007	*C. glabrata*
64	8	32	0.007	*C. glabrata*
64	0. 25	32	0.007	*C. glabrata*
64	8	8	0.007	*C. glabrata*
64	0. 25	32	0.007	*C. glabrata*
64	1	32	0.007	*C. glabrata*
64	0. 5	32	0.007	*C. glabrata*
64	2	32	0.007	*C. glabrata*
64	0.25	32	0.007	*C. glabrata*
64	2	8	0.007	*C. glabrata*
64	1	32	0.007	*C. glabrata*
64	2	32	0.007	*C. glabrata*
64	0.5	32	0.007	*C. glabrata*
64	4	32	0.007	*C. glabrata*
64	0. 5	32	0.007	*C. glabrata*
16	8	16	0.007	*C. albicans*
64	8	16	0.007	*C. albicans*
64	4	16	0.007	*C. albicans*
64	0.125	0.03	0.007	*C. albicans*
64	0.03	16	0.007	*C. albicans*
64	0.5	0.03	0.007	*C. albicans*
64	4	0.03	0.007	*C. albicans*
64	0.03	16	0.007	*C. albicans*
64	4	16	0.007	*C. albicans*
64	0.007	16	0.007	*C. albicans*
64	8	32	0.007	*C. albicans*
64	0.25	32	0.007	*C. albicans*
64	8	32	0.007	*C. albicans*
0.125	0.03	0.06	0.007	*C. albicans*
64	1	32	0.007	*C. albicans*
64	2	32	0.007	*C. albicans*
64	0.03	32	0.007	*C. albicans*
64	2	32	0.007	*C. albicans*
64	2	32	0.007	*C. albicans*
64	0.5	32	0.007	*C. albicans*
64	16	16	0.007	*C. krusei*
64	0.03	16	0.007	*C. krusei*
64	16	16	0.007	*C. krusei*
64	2	32	0.007	*C. krusei*
64	0.03	0.03	0.007	*C. kefyr*
0.06	0.03	16	0.007	*C. kefyr*
64	0.03	16	0.007	*C. kefyr*
4	0.03	32	0.007	*C. kefyr*
64	0.03	16	0.007	*C. tropicalis*

### 3.4. Drug Susceptibility Pattern of Isolated *Candida* Species to Antifungal Drugs

The findings of the present study reveal significant resistance and susceptibility patterns among different *Candida* species. As shown in the composite Table 5, all 38 *C. glabrata* isolates (100%) demonstrated complete resistance to both fluconazole and amphotericin B, while showing remarkable susceptibility to caspofungin (37 isolates, 97.4%). For itraconazole, an intermediate resistance pattern was observed with 30 resistant isolates (78.9%) and 8 susceptible isolates (21.1%). Among 20 *C. albicans* isolates, we observed high resistance to fluconazole (19 isolates, 95%) and amphotericin B (16 isolates, 80%). Notably, this group showed 100% susceptibility to caspofungin. The resistance pattern to itraconazole was more balanced in this group (11 resistant isolates, 55% vs. 9 susceptible isolates, 45%). All four *C. krusei* isolates (100%) also exhibited complete resistance to fluconazole and amphotericin B, while demonstrating 100% susceptibility to caspofungin. The itraconazole resistance pattern in this group was similar to *C. albicans* (three resistant isolates, 75% vs. one susceptible isolate, 25%). Interesting findings were observed in four *C. kefyr* isolates, which showed complete susceptibility (100%) to itraconazole and caspofungin. In this group, resistance to fluconazole (three isolates, 75%) and amphotericin B (three isolates, 75%) was at moderate levels. The single *C. tropicalis* isolate studied showed complete resistance to fluconazole and amphotericin B (100%) along with complete susceptibility to itraconazole and caspofungin (100%).

**Table 5:** Drug susceptibility pattern of 67 *Candida* isolates to antifungal agents.

Antifungal drug	Species				
	*C. glabrata*	*C. albicans*	*C. krusei*	*C. kefyr*	*C. tropicalis*
**Fluconazole**	Resistant isolates: 38 (100%)	Resistant isolates: 19 (95%)	Resistant isolates: 4 (100%)	Resistant isolates: 3 (75%)	Resistant isolates: 1 (100%)
	Susceptible isolates: —	Susceptible isolates: 1 (5%)	Susceptible isolates: —	Susceptible isolates: 1 (25%)	Susceptible isolates: —

**Itraconazole**	Resistant isolates: 30 (78.9%)	Resistant isolates: 11 (55%)	Resistant isolates: 3 (75%)	Resistant isolates: —	Resistant isolates: —
	Susceptible isolates: 8 (21.1%)	Susceptible isolates: 9 (45%)	Susceptible isolates: 1 (25%)	Susceptible isolates: 4 (100%)	Susceptible isolates: 1 (100%)

**Caspofungin**	Resistant isolates: 1 (2.6%)	Resistant isolates: —	Resistant isolates: —	Resistant isolates: —	Resistant isolates: —
	Susceptible isolates: 37 (97.94%)	Susceptible isolates: 20 (100%)	Susceptible isolates: 4 (100%)	Susceptible isolates: 4 (100%)	Susceptible isolates: 1 (100%)

**Amphotericin B**	Resistant isolates: 38 (100%)	Resistant isolates: 16 (80%)	Resistant isolates: 4 (100%)	Resistant isolates: 3 (75%)	Resistant isolates: 1 (100%)
	Susceptible isolates: —	Susceptible isolates: 4 (20%)	Susceptible isolates: —	Susceptible isolates: 1 (25%)	Susceptible isolates: —

*Note:* Data are based on 67 isolates that were successfully tested for antifungal susceptibility (38 *C. glabrata*, 20 *C. albicans*, 4 *C. krusei*, 4 *C. kefyr*, and 1 *C. tropicalis*).

## 4. Discussion

IC in the PICU remains a serious hospital‐acquired infection, associated with increased morbidity, mortality, and hospital costs, and the *Candida* species involved varies considerably [16]. Risk factors for Candida infections have been reported to include the presence of central venous catheters (CVCs), the presence of endotracheal tubes, long‐term use of broad‐spectrum antibiotics, prolonged PICU stay, parenteral nutrition, gastrointestinal‐surgical trauma, corrective surgery for congenital heart disease, previous *Candida* colonization at multiple sites, and underlying malignancy [16]. In this study, a total of 97 clinical samples were initially collected from children and infants hospitalized in different departments of Tabriz Children′s Hospital over a 1‐year period. All 97 isolates underwent molecular identification using ITS sequencing. For the antifungal susceptibility testing phase, 67 isolates were successfully recovered and evaluated. These 67 isolates comprised: *C. glabrata* (38 cases), *C. albicans* (20 cases), *C. krusei* (4 cases), *C. kefyr* (4 cases), and *C. tropicalis* (1 case). The remaining 30 isolates were excluded from susceptibility testing due to viability issues upon subculture. In this study, the rate of nonalbicans *Candida* was 56.7%, whereas in previous studies, it was reported as 35.8% [17] and 78.2% [18]. The increase in nonalbicans species due to long‐term use of antifungals leads to higher levels of resistance of *Candida* strains to antifungal drugs [19]. Notably, in our study, the most common species isolated in clinical specimens was *C. glabrata*, which can be a serious threat due to its resistance to common antifungal agents.

The optimal antifungal agent and the optimal dose are unclear, especially for infants and young children with IC in the PICU; therefore, treatment of IC in the PICU should follow the basic principle of treatment in all patient groups, which is early identification and elimination of the possible source of infection [20, 21]. The optimal drug of choice for this pediatric population has not yet been identified. The choice should be based on local epidemiology (rate of azole resistance among nonalbicans species), previous colonization, critical conditions, and previous exposure to azoles. Vegiazzi et al. suggested that antifungal agents such as polyenes and echinocandins are preferred as first‐line treatment options in critically ill patients [22]. According to the guidelines, fluconazole is recommended for patients with mild to moderate disease and no prior exposure to azoles, and echinocandins or lipid‐based polyenes are recommended for patients with moderate to severe disease or prior exposure to azoles. In this study, the range of fluconazole MICs was 0.06–64 *μ*g/mL. Most strains were resistant to fluconazole in most cases, and only 3% of isolates were susceptible to fluconazole. The results of this study were similar to those of Colomer et al., who studied *Candida* isolates from infants admitted to the intensive care unit and found that only 4.3% of isolates were susceptible to fluconazole [23]. The results of this study were different from the study by Badiei et al. In their study, which investigated the susceptibility of *Candida* in patients with immunodeficiency disorders, it was reported that only 10.5% of *C. albicans* isolates were resistant [19]. In a study by Salehi et al., *C. albicans* was 100% susceptible to fluconazole, and 50% of *C. glabrata* isolates were resistant to fluconazole [24].

Correct epidemiology and transmission routes of nosocomial *Candida* infections and the establishment of specific control measures are needed to see a reduction in the incidence of these infections. In this context, molecular detection of *Candida* species is essential for a better understanding of the epidemiology of these infections and appropriate prevention strategies. Over the past two decades, molecular typing methods have been developed, which are of great importance in identifying infectious strains for epidemiological studies and determining the microbial population structure and genetic diversity within a species. Various typing methods have been investigated to determine the molecular characteristics of *Candida* species [25]. In 2017, Jafari et al. conducted a study to identify *C. albicans* and *C. dubliniensis*. These two species cannot be distinguished by conventional phenotypic methods. First, they isolated 100 *Candida* strains using phenotypic methods, and then used the PCR‐RFLP method for more accurate identification. According to the results, all 100 strains were related to *C. albicans*. This study showed that *C. dubliniensis* did not play a role in vaginal candidiasis in the patients studied [26].

In a descriptive study conducted in 2015–2016, Kord et al. examined vaginal discharge samples from 189 women with clinical symptoms of vulvovaginitis and cultured them on SDA for definitive diagnosis. *Candida* isolates were identified by molecular PCR‐RFLP, and of these 189 samples, 108 samples had positive *Candida* colonies that were examined and studied. The results of species identification and identification of *Candida* colonies by PCR‐RFLP indicated 5 *Candida* species, of which 84 were *C. albicans*, 10 were *C. glabrata*, 4 were *C. kefir*, 1 *C. parapsilosis*, and 1 was *C. hemotropicalis*. In addition, 8 of the samples had two *Candida* species [27]. Another study by Sandra et al. in 2004 was aimed at identifying *Candida* species by phenotypic and PCR methods. Samples were collected from the oral mucosa of 38 adult Brazilian male and female patients with oral candidiasis. A total of 24 out of 38 strains were able to produce germinal tubes. Chlamydoconidia production was observed in 28 cases, and 24 of the tested strains showed green colonies on CHROMagar medium, indicating *C. albicans* or *C. dubliniensis*. To confirm these tests, a carbohydrate absorption test was used on all 38 strains, and 24 strains indicated *C. albicans* and *C. dubliniensis*. A total of eight strains that were neither able to form germinal tubes nor able to produce chlamydoconidia were identified as *C. krusei* by *Candida* CHROMagar and carbohydrate absorption test [28]. Therefore, in the present study, molecular methods such as PCR were used to more reliably determine *Candida* species. Although phenotypic methods are somewhat simple, easy, and inexpensive, their results are general and, in some cases, unreliable. On the other hand, the use of these methods is usually time‐consuming or often unable to distinguish *C. albicans* and *C. dubliniensis*, and obtaining false positive or negative results is inevitable because, in this type of study, there is a possibility of creating a switching phenotype in Candida species. Therefore, molecular techniques have been considered in newer methods. Of course, genotypic methods have always been more reliable. Although the use of these techniques requires more cost than phenotypic methods, their accuracy, precision, and speed are undeniable [29]. Although ITS sequencing provided reliable species‐level identification in the present study, we acknowledge that constructing phylogenetic trees comparing our isolates with global reference strains could offer additional insights. Such analysis would enable: (1) identification of potential cryptic species that may not be distinguishable by ITS amplicon size or conventional sequencing alone; (2) determination of genetic relationships between local isolates and global strains; (3) tracking of potential transmission routes and outbreak strains; and (4) detection of strain‐level variations that might correlate with antifungal resistance patterns or virulence potential. However, the ITS region, although excellent for species identification, has limitations for fine‐scale phylogenetic analysis due to its high conservation within species. Future studies employing multilocus sequence typing (MLST) or whole‐genome sequencing approaches would enable more robust phylogenetic comparisons and potentially identify emerging cryptic species or strain‐specific genetic markers associated with antifungal resistance in our pediatric population.

## 5. Conclusion

Neonates and children are more vulnerable to invasive fungal infections due to biological and immunological differences. Recent years have seen a sharp rise in such infections, yet data on *Candida* species distribution and resistance in pediatric settings remain limited. This study found that *C. glabrata* was the most common species among 97 isolates, indicating a shift from *C. albicans*. Antifungal susceptibility testing of 67 viable isolates revealed that while fluconazole showed low effectiveness, caspofungin demonstrated high efficacy, suggesting its potential as a first‐line treatment. PCR proved faster and more reliable than traditional phenotypic methods for species identification. These findings emphasize the need for accurate diagnostics, targeted antifungal therapy, and ongoing surveillance to combat resistance and improve outcomes in pediatric patients. Further clinical studies are essential to refine treatment strategies.

## Author Contributions

**A.D., N.A.,** and **B.N.H.:** study conception and design; **K.H., T.M.,** and **M.M.S.:** carried out the experiments; **M.A.R.** and **M.B.H.:** samples collection; **K.H.:** drafting and writing of the manuscript.

## Funding

No funding was received for this manuscript.

## Ethics Statement

This research was managed based on regulatory and ethics committee approval in Iran, and the ethical approval of this research was obtained from the Ethics Committee of Tabriz University of Medical Sciences, Tabriz, Iran. Written informed consent was obtained from all living patients at the Children′s Medical and Training Center Hospital at the Tabriz University of Medical Sciences site. The experimental protocol conformed to the National Institutes of Health guidelines and was approved by the Ethical Committee of Tabriz University of Medical Sciences, Tabriz, Iran (Ethical code:) as a PharmD Theses (#Grant No: 66631,66904, IR.TBZMED.REC.1400.408, IR.TBZMED.REC.1400.041).

## Conflicts of Interest

The authors declare no conflicts of interest.

## Data Availability

All data generated or analyzed during this study are included in this article.

## References

[1] Rosenthal V. D. , Bijie H. , Maki D. G. , Mehta Y. , Apisarnthanarak A. , Medeiros E. A. , Leblebicioglu H. , Fisher D. , Álvarez-Moreno C. , Khader I. A. , del Rocío González Martínez M. , Cuellar L. E. , Navoa-Ng J. A. , Abouqal R. , Guanche Garcell H. , Mitrev Z. , Pirez García M. C. , Hamdi A. , Dueñas L. , Cancel E. , Gurskis V. , Rasslan O. , Ahmed A. , Kanj S. S. , Ugalde O. C. , Mapp T. , Raka L. , Yuet Meng C. , Thu le T. A. , Ghazal S. , Gikas A. , Narváez L. P. , Mejía N. , Hadjieva N. , Gamar Elanbya M. O. , Guzmán Siritt M. E. , Jayatilleke K. , and INICC members , International Nosocomial Infection Control Consortium (INICC) Report, Data Summary of 36 Countries, for 2004-2009, American Journal of Infection Control. (2012) 40, no. 5, 396–407, 10.1016/j.ajic.2011.05.020, 21908073. 21908073

[2] Shaikh J. M. , Devrajani B. R. , Shah S. Z. , Akhund T. , and Bibi I. , Frequency, Pattern and Etiology of Nosocomial Infection in Intensive Care Unit: An Experience at a Tertiary Care Hospital, Journal of Ayub Medical College, Abbottabad. (2008) 20, no. 4, 37–40. 19999200

[3] Khodavaisy S. , Mohammadi F. , Fesharaki S. H. , Samieeifard S. H. , Solimani P. , and Badali H. , Candida Biofilms and Their Less Susceptibility to Antifungal Drugs, Clinical Excellence. (2014) 3, 58–71.

[4] Taheri Sarvtin M. , Kamali M. , and Yazdani J. , A Review on the Risk Factors, Presentations and Treatment of Candidemia, Journal of Jiroft University of Medical Sciences. (2016) 2, no. 2, 55–60.

[5] Parahym A. M. R. D. C. , Melo L. R. B. , Morais V. L. L. D. , and Neves R. P. , Candidiasis in Pediatric Patients With Cancer Interned in a University Hospital, Brazilian Journal of Microbiology. (2009) 40, no. 2, 321–324, 10.1590/S1517-83822009000200020. 24031365 PMC3769713

[6] Wander K. , O′Connor K. , and Shell-Duncan B. , Expanding the Hygiene Hypothesis: Early Exposure to Infectious Agents Predicts Delayed-Type Hypersensitivity to Candida Among Children in Kilimanjaro, PLoS One. (2012) 7, no. 5, e37406, 10.1371/journal.pone.0037406, 22616000. 22616000 PMC3355133

[7] Belet N. , Ciftçi E. , Aysev D. , Güriz H. , Uysal Z. , Taçyildiz N. , Atasay B. , Doğu F. , Kendirli T. , Kuloğlu Z. , Ince E. , and Doğru U. , Invasive Candida Infections in Children: The Clinical Characteristics and Species Distribution and Antifungal Susceptibility of Candida spp, Turkish Journal of Pediatrics. (2011) 53, no. 5, 489–498, 22272448. 22272448

[8] Tamo S. B. , Candida Infections: Clinical Features, Diagnosis and Treatment, Infectious Diseases and Clinical Microbiology. (2020) 2, no. 2, 91–102, 10.36519/idcm.2020.0006.

[9] Williams D. W. , Wilson M. J. , Lewis M. A. , and Potts A. J. , Identification of Candida Species by PCR and Restriction Fragment Length Polymorphism Analysis of Intergenic Spacer Regions of Ribosomal DNA, Journal of Clinical Microbiology. (1995) 33, no. 9, 2476–2479, 10.1128/jcm.33.9.2476-2479.1995, 7494052. 7494052 PMC228446

[10] Zaoutis T. E. , Prasad P. A. , Localio A. R. , Coffin S. E. , Bell L. M. , Walsh T. J. , and Gross R. , Risk Factors and Predictors for Candidemia in Pediatric Intensive Care Unit Patients: Implications for Prevention, Clinical Infectious Diseases. (2010) 51, no. 5, e38–e45, 10.1086/655698, 20636126. 20636126 PMC3753770

[11] Eddouzi J. , Parker J. E. , Vale-Silva L. A. , Coste A. , Ischer F. , Kelly S. , Manai M. , and Sanglard D. , Molecular Mechanisms of Drug Resistance in Clinical Candida Species Isolated From Tunisian Hospitals, Antimicrobial Agents and Chemotherapy. (2013) 57, no. 7, 3182–3193, 10.1128/AAC.00555-13, 23629718. 23629718 PMC3697321

[12] Cowen L. E. , Sanglard D. , Howard S. J. , Rogers P. D. , and Perlin D. S. , Mechanisms of Antifungal Drug Resistance, Cold Spring Harbor Perspectives in Medicine. (2015) 5, no. 7, a019752, 10.1101/cshperspect.a019752, 25384768. PMC4484955 25384768

[13] Cavalheiro M. and Teixeira M. C. , Candida Biofilms: Threats, Challenges, and Promising Strategies, Frontiers in Medicine. (2018) 5, 10.3389/fmed.2018.00028, 29487851. PMC5816785 29487851

[14] Marak M. B. and Dhanashree B. , Antifungal Susceptibility and Biofilm Production ofCandidaspp. Isolated From Clinical Samples, International Journal of Microbiology. (2018) 2018, no. 1, 5, 10.1155/2018/7495218. PMC6199855 30405717

[15] McGinnis M. R. and Tyring S. K. , Introduction to Mycology, Medical Microbiology, 1996, 4, 4th edition, University of Texas Medical Branch at Galveston. 21413300

[16] Pana Z. D. , Roilides E. , Warris A. , Groll A. H. , and Zaoutis T. , Epidemiology of Invasive Fungal Disease in Children, Journal of the Pediatric Infectious Diseases Society. (2017) 6, no. 1 Supplement, S3–S11, 10.1093/jpids/pix046, 28927200. 28927200 PMC5907880

[17] Lopes M. , Barros R. , Peres I. , Serelha M. , Neto M. T. , Cabrita J. , and Freitas G. , Surveillance of Nosocomial Fungal Infections in a Portuguese Paediatric Hospital: Incidence and Risk Factors, Journal de Mycologie Médicale. (2006) 16, no. 4, 212–219, 10.1016/j.mycmed.2006.08.004.

[18] Pasqualotto A. C. , Nedel W. L. , Machado T. S. , and Severo L. C. , A 9-Year Study Comparing Risk Factors and the Outcome of Paediatric and Adults With Nosocomial Candidaemia, Mycopathologia. (2005) 160, no. 2, 111–116, 10.1007/s11046-005-3452-1, 16170605. 16170605

[19] Badiee P. and Alborzi A. , Susceptibility of Clinical Candida Species Isolates to Antifungal Agents by E-Test, Southern Iran: A Five Year Study, Iranian Journal of Microbiology. (2011) 3, no. 4, 183–188, 22530086. 22530086 PMC3330181

[20] Pappas P. G. , Kauffman C. A. , Andes D. R. , Clancy C. J. , Marr K. A. , Ostrosky-Zeichner L. , Reboli A. C. , Schuster M. G. , Vazquez J. A. , Walsh T. J. , Zaoutis T. E. , and Sobel J. D. , Clinical Practice Guideline for the Management of Candidiasis: 2016 Update by the Infectious Diseases Society of America, Clinical Infectious Diseases. (2016) 62, no. 4, e1–e50, 10.1093/cid/civ933, 26679628. 26679628 PMC4725385

[21] Hope W. , Castagnola E. , Groll A. H. , Roilides E. , Akova M. , Arendrup M. C. , Arikan-Akdagli S. , Bassetti M. , Bille J. , Cornely O. A. , Cuenca-Estrella M. , Donnelly J. P. , Garbino J. , Herbrecht R. , Jensen H. E. , Kullberg B. J. , Lass-Flörl C. , Lortholary O. , Meersseman W. , Petrikkos G. , Richardson M. D. , Verweij P. E. , Viscoli C. , Ullmann A. J. , and ESCMID Fungal Infection Study Group , ESCMID* Guideline for the Diagnosis and Management of Candida Diseases 2012: Prevention and Management of Invasive Infections in Neonates and Children Caused by Candida spp, Clinical Microbiology and Infection. (2012) 18, 38–52, 10.1111/1469-0691.12040, 23137136. 23137136

[22] Vogiatzi L. , Ilia S. , Sideri G. , Vagelakoudi E. , Vassilopoulou M. , Sdougka M. , Briassoulis G. , Papadatos I. , Kalabalikis P. , Sianidou L. , and Roilides E. , Invasive Candidiasis in Pediatric Intensive Care in Greece: A Nationwide Study, Intensive Care Medicine. (2013) 39, no. 12, 2188–2195, 10.1007/s00134-013-3057-y, 23942859. 23942859

[23] Colomer B. F. , Cotallo G. C. , Sastre J. L. , and Castrillo , Neonatal Invasive Candidiasis in Spain: An Epidemiological Study From the “Grupo de Hospitales Castrillo”, American Journal of Perinatology. (2016) 33, no. 01 Supplement, 10.1055/s-0036-1592383.

[24] Salehi F. , Esmaeili M. , and Mohammadi R. , Isolation of Candida Species From Gastroesophageal Lesions Among Pediatrics in Isfahan, Iran: Identification and Antifungal Susceptibility Testing of Clinical Isolates by E-Test, Advanced Biomedical Research. (2017) 6, no. 1, 10.4103/2277-9175.213662. PMC5590398 28904931

[25] Bougnoux M.-E. , Aanensen D. M. , Morand S. , Théraud M. , Spratt B. G. , and d’Enfert C. , Multilocus Sequence Typing of Candida albicans: Strategies, Data Exchange and Applications, Infection, Genetics and Evolution. (2004) 4, no. 3, 243–252, 10.1016/j.meegid.2004.06.002. 15450203

[26] Jafari M. , Salari S. , Pakshir K. , and Zomorodian K. , Exoenzyme Activity and Possibility Identification of Candida dubliniensis Among Candida albicans Species Isolated From Vaginal Candidiasis, Microbial Pathogenesis. (2017) 110, 73–77, 10.1016/j.micpath.2017.06.024, 28642006. 28642006

[27] Kord Z. , Fata A. , and Zarrinfar H. , Molecular Identification of Candida Species Isolated From Patients With Vulvovaginitis for the First Time in Mashhad, Iranian Journal of Obstetrics, Gynecology and Infertility. (2017) 20, no. 4, 50–57.

[28] Marinho S. A. , Teixeira A. B. , Santos O. S. , Cazanova R. F. , Ferreira C. A. S. , Cherubini K. , and Oliveira S. D. , Identification of Candida spp. by Phenotypic Tests and PCR, Brazilian Journal of Microbiology. (2010) 41, no. 2, 286–294, 10.1590/S1517-83822010000200004, 24031493. 24031493 PMC3768677

[29] Saha R. , das das S. , Kumar A. , and Kaur I. R. , Pattern of Candida Isolates in Hospitalized Children, Indian Journal of Pediatrics. (2008) 75, no. 8, 858–860, 10.1007/s12098-008-0159-6. 18769898

